# Evolution of *Wolbachia* reproductive and nutritional mutualism: insights from the genomes of two novel strains that double infect the pollinator of dioecious *Ficus hirta*

**DOI:** 10.1186/s12864-023-09726-2

**Published:** 2023-11-01

**Authors:** Wanzhen Liu, Xue Xia, Ary A. Hoffmann, Yamei Ding, Ji-Chao Fang, Hui Yu

**Affiliations:** 1 Key Laboratory of Plant Resource Conservation and Sustainable Utilization, South China Botanical Garden, The Chinese Academy of Sciences, Guangzhou, 510650 China; 2 Institute of Plant Protection, Jiangsu Key Laboratory for Food and Safety-State Key Laboratory Cultivation Base of Ministry of Science and Technology, Jiangsu Academy of Agricultural Sciences, Nanjing, 210014 China; 3 School of BioSciences, Bio21 Institute, University of Melbourne, Parkville, VIC Australia

**Keywords:** *Wolbachia*-pollinator-host plant interactions, Mutualism, Cytoplasmic incompatibility, Biotin, Genome

## Abstract

*Wolbachia* is a genus of maternally inherited endosymbionts that can affect reproduction of their hosts and influence metabolic processes. The pollinator, *Valisia javana*, is common in the male syconium of the dioecious fig *Ficus hirta*. Based on a high-quality chromosome-level *V. javana* genome with PacBio long-read and Illumina short-read sequencing, we discovered a sizeable proportion of *Wolbachia* sequences and used these to assemble two novel *Wolbachia* strains belonging to supergroup A. We explored its phylogenetic relationship with described *Wolbachia* strains based on MLST sequences and the possibility of induction of CI (cytoplasmic incompatibility) in this strain by examining the presence of *cif* genes known to be responsible for CI in other insects. We also identified mobile genetic elements including prophages and insertion sequences, genes related to biotin synthesis and metabolism. A total of two prophages and 256 insertion sequences were found. The prophage WOjav1 is cryptic (structure incomplete) and WOjav2 is relatively intact. IS5 is the dominant transposon family. At least three pairs of type I *cif* genes with three copies were found which may cause strong CI although this needs experimental verification; we also considered possible nutritional effects of the *Wolbachia* by identifying genes related to biotin production, absorption and metabolism. This study provides a resource for further studies of *Wolbachia*-pollinator-host plant interactions.

**Supplementary Information:**

The online version contains supplementary material available at 10.1186/s12864-023-09726-2.

## Background

*Wolbachia* is a genus of maternally inherited intracellular endosymbiotic bacteria belonging to the order Rickettsiales and is estimated to be distributed in ca. 44% of arthropods, and 66% of insects, and also shows mutualistic symbiosis in nematodes [1–3]. *Wolbachia* is classified as a single species, *Wolbachia pipientis*, divided into supergroups A to U, with supergroups A and B infecting arthropods exclusively, based on the phylogenetic analysis using the 16 S rRNA, *wsp*, *gatB*, *coxA*, *hcpA*, *fbpA*, and *ftsZ* markers [4–7]. *Wolbachia* is transmitted mainly via vertical transmission from mother to offspring with high fidelity, but also can be transmitted across different taxa by host shift, especially for supergroups A and B occurring between both phylogenetically close and more distant host species [4, 8, 9].

To increase transmission and propagation, *Wolbachia* can impact the reproductive system of arthropod and nematode species using diverse methods, such as cytoplasmic incompatibility (CI), feminization of genetic males, parthenogenesis, and male-killing [10–13]. CI is the most common reproductive manipulation effect induced by *Wolbachia*, and can be used as an important means to control arboviruses by release of CI including males [14] or CI driven replacement of uninfected mosquito populations [15, 16]. Co-expression of the prophage WO genes *cifA* and *cifB* in *Wolbachia* can cause a CI phenotype, which can be rescued by maternal *cifA* expression [17]. Phylogenetic analysis of the *cif* protein, which is encoded by the *cif* gene, shows that the evolution of *cifA* and *cifB* is related to each other. Both *cifA* and *cifB* can be divided into five distinct phylogenetic clades as type I-V [18–20].

*Wolbachia* can also exert an influence on numerous processes in the host, including immune, behavioral, and metabolic processes [21], which may be related to their occupation of host cells and reproductive tissues [22]. *Wolbachia* may be beneficial in insects by protecting them from pathogenic viral infection and increasing host fitness and fecundity [23–26], although there can also be fitness costs particularly in transgenic *Wolbachia* infections [27]. In nematodes and *Cimex lectularius*, *Wolbachia* functions as an obligatory mutualistic endosymbiont [20, 28] but there are also *Wolbachia* infections with few phenotypic effects on host [27].

The obligate mutualism of figs and fig-pollinating wasps has been one of the classic models used for testing theories of co-evolution and cospeciation due to the high species-specificity of these relationships [29]. Figs (*Ficus*, (Moraceae) include around 750 species worldwide, distributed in the tropics and subtropics [30, 31]. Figs and fig-pollinating wasps (Agaonidae; Chalcidoidea; Hymenoptera) are obligate mutualists that have coevolved for over ~ 75 million years [32, 33]. Except for pollinators, numerous non-pollinating fig wasps feed on fig tissue or other fig wasps [34–36] and some lay eggs outside the syconium with remarkable long ovipositors. In most fig species, each syconium contains 50–500 wasps of a few different species developing in close proximity [37]. As such, this is a useful system for investigating the impact of ecological interactions on *Wolbachia* transmission and persistence.

More than half of the pollinating fig wasp species from North America, Australia, Asia, and Africa are infected with *Wolbachia* [9, 37–40], which is high compared to many other insects [40]. The comparison of endosymbiont diversity between female and male syconium in the dioecious *Ficus hirta* using high-throughput sequencing and biological databases showed *Wolbachia* is dominant in the male syconium infested by fig wasps in contrast to the female syconium, because the latter has no galled flowers for fig wasp larvae [41]. Moreover, genomes of *Wolbachia* from three pollinating fig wasp species (*Ceratosolen solmsi, Kradibia gibbosae*, and *Wiebesia pumilae*) have been classified into supergroup A and only one cryptic (incomplete structure) WO prophage bearing CI related genes has so far been found, whereas *Wolbachia* with cryptic prophages usually come with least one intact WO prophage consisting of gene sequences of the head, baseplate, and tail modules, through which the prophage could form intact virions [42].

To recover the *Wolbachia* genome, we used a high-quality chromosome-level genome for *V. javana* Mayr (Agaonidae, Chalcidoidea, Hymenoptera), formerly known as *Blastophaga javana* Mayr, assembled using a combination of PacBio long-read sequencing and Illumina short read sequencing [43]. From the *V. javana* genomic data, we discovered a significant fraction of *Wolbachia* genome sequence and used these to assemble two novel *Wolbachia* strains. We explored their phylogenetic relationship with described *Wolbachia* strains based on MLST and explored the possibility that strains could induce CI by exploring the presence of genes responsible for CI. Furthermore, to explore the possibility of nutritional mutualism, we identified mobile genetic elements (MGEs) including prophages and insertion sequences, genes related to biotin (vitamin B7) synthesis and metabolism, and *cif* homologous genes in the mixed genome. This genome assembly will serve as a useful resource for further studies of *Wolbachia*-pollinator-host interactions, comparative genomics, and coevolution.

## Results

### *V. javana* double infection with two novel *Wolbachia* strains

Based on the length of the *Wolbachia* mixed genome of *V. javana* and the number of fragments, we selected the sequence with a length of 2,352,926 bp (2.24 Mb) as the final one (Table 1). After polishing using Illumina data, a genomic sequence with a length of 2,352,827 bp was obtained. The GC content of the genome was 35.0%, and the number of CDS (protein coding sequence) and RNAs were 2,825 and 62, respectively (Table 2). Since the genome size of the mixed genome is much larger than the known *Wolbachia* (Table S1) and the genomes are difficult to separate, MLST (multilocus sequence typing) sequences from the infected *w*Mel strain *Drosophila melanogaster* (ST = 1), were used to search for five MLST housekeeping genes. There were at least two sets of MLST sequences with unique allelic profile combinations in the genomes (Table 3). However, none of these combinations belong to any known *Wolbachia* strains, so they are identified as two novel strains called *w*Jav1 and *w*Jav2.

**Table 1:** Different reads were screened by BLASR and assembled with different parameters of Flye

BLASR parameter*	Total length of reads (bp)	Flye parameter*	Genome size after assembly (bp)	Number of Fragments
Identity ≥ 60%, maxscore≤-250	1,024,923,876	minimum overlap between reads ≥ 1000 bp	53,393,107	1561
		minimum overlap between reads ≥ 3000 bp	53,568,170	1501
		minimum overlap between reads ≥ 5000 bp	53,930,492	1476
Identity ≥ 60%, maxscore≤-300	639,517,259	minimum overlap between reads ≥ 1000 bp	1,734,532	70
		minimum overlap between reads ≥ 3000 bp	2,258,584	55
		minimum overlap between reads ≥ 5000 bp	2,352,926	50

Note: * The asterisk indicates that the other command parameters of BLASR are the same. The commands for BLASR are available in Table S9

**Table 2:** Genome statistics of *Wolbachia* in *Valisia javana*

Parameters	*w*Jav1 + *w*Jav2
Host	*Valisia javana*
Infection type	Multiple infection, two different strains
Total size (bp)	2,352,827
Number of contigs	50
GC content (%)	35.0
Number of CDS	2,825
Number of RNAs	62

**Table 3:** MLST sequence of *Wolbachia* in *Valisia javana*

Query seqence	Identity	Length	Evalue	Housekeeping gene name (Allele)
Figure 953.597.peg.158	98.113	371	0	***gatB*** [77]
Figure 953.597.peg.671	99.317	439	0	*fbpA* [62]
Figure 953.597.peg.810	99.507	406	0	***coxA*(1**) 1 difference found. ^51^T → ^576^G
Figure 953.597.peg.874	99.754	406	0	*coxA* [1]
Figure 953.597.peg.1067	96.305	406	0	***coxA*** [37]
Figure 953.597.peg.1260	97.732	441	0	*hcpA* [60]
Figure 953.597.peg.1667	99.772	439	0	*ftsZ* [3]
Figure 953.597.peg.2329	100	371	0	***gatB***[1]
Figure 953.597.peg.2642	99.772	439	0	*ftsZ* [3]
Figure 953.597.peg.2669	99.317	439	0	*fbpA* [62]

**Note** Genes with two sequence accession numbers are indicated by boldface type. The query sequence can refer to Table S8. “Allele” is a serial number for specific sequences of housekeeping gene

### Identification of *w* Jav1 and *w* Jav2

**Fig. 1:**
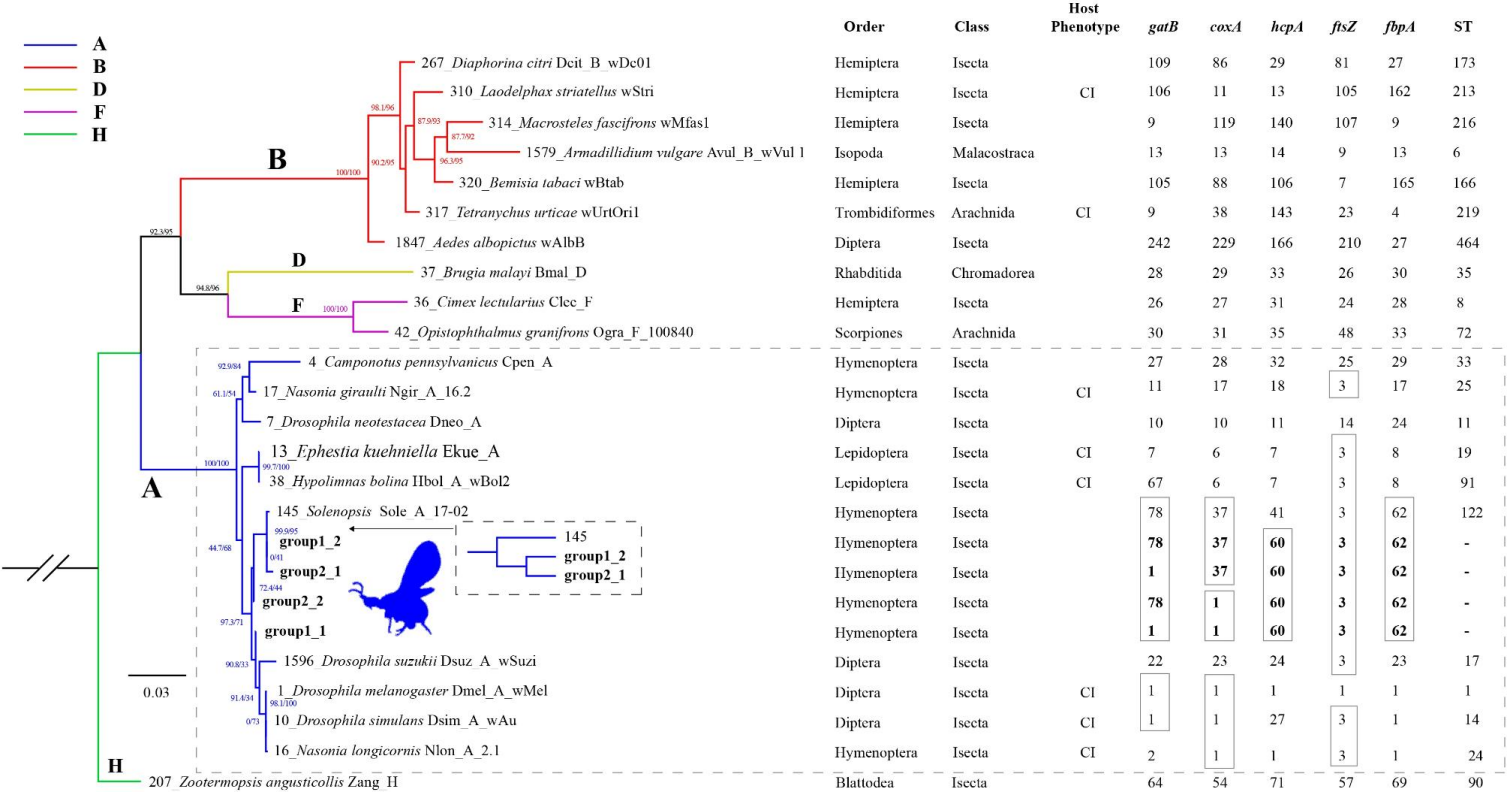
Phylogenetic analysis of MLST data for 21 species of *Wolbachia* belonging to different supergroups based on maximum-likelihood. SH-aLRT (SH-like approximate likelihood ratio test) and UFBoot (ultrafast bootstrap approximation) support values are given on nodes. The corresponding sample ids, hosts, strains, and colors represent different supergroups of *Wolbachia* strains can be found in Table S2. MLST groups can be found in Table S8

The MLST sequences in *w*Jav1 and *w*Jav2 can be divided into two possible groups (Table 4) and are unique when compared with the known MLST sequences in the pubMLST database. The *w*Jav1 and *w*Jav2 strains have different profiles of *gatB* and *coxA* (Fig. 1; Table 4). The Sole_A_17 − 02 and *w*Jav2 strains only differ for the *hcpA* gene (Table 4), suggesting that the wJav strains are linked to this strain, perhaps due to horizontal transmission among hosts. The *gatB* and *coxA* sequences can be divided into two *w*Jav types (Fig. 1). Based on *hcpA* and *ftz* sequences, *V. javana Wolbachia* are the most similar in the PubMLST to the white-backed planthopper *Sogatella furcifera* (Sfur_A_YN4) and *Megastigmus* sp.3 (Mspi_A_wspe) (Fig. 1, Table S2). The former is the main pest on rice paddies throughout Asia [44], and the latter is a serious pest of conifer species with four basic feeding types [45].

**Table 4:** Different combinations of MLST allelic profiles for *w*Jav1 and *w*Jav2, and unique MLST allelic profiles for the five closed-related species

Group	Strains	*gatB*	*coxA*	*hcpA*	*ftsZ*	*fbpA*	ST	Host species	Supergroup
Group 1	*w*Jav1	1	1	60	3	62		*Valisia javana*	
	*w*Jav2	78	37	60	3	62		*Valisia javana*	
Group 2	*w*Jav1	1	37	60	3	62		*Valisia javana*	
	*w*Jav2	78	1	60	3	62		*Valisia javana*	
	A_M11He	54	52	62	3	62	83	unknown	A
	Sole_A_17 − 02	78	37	41	3	62	122	*Solenopsis*	A
	Mchi_A	78	37	90	3	62	123	unknown	A
	A_NY_Calyp150744c	78	37	62	3	62	132	unknown	A
	A_Pan_Droso150749b	93	37	93	3	62	133	unknown	A

We randomly selected 21 *Wolbachia* MLST sequences (Table S2) belonging to different supergroups (A, B, D, F, H) and different hosts to construct an evolutionary tree by the maximum likelihood (ML) method. The ML tree showed that both *w*Jav1 and *w*Jav2 belong to supergroup A and are closely related to each other (Fig. 1). The most similar strain of *w*Jav2 of group 1 is Sole_A_17 − 02 infecting the fire ant (*Solenopsis*, Apocrita, Hymenoptera) which belongs to supergroup A (Fig. 1, Table S2). The relationship between the mixed genomes and the remaining 165 *Wolbachia* genomes was further evaluated by average nucleotide identity (ANI) (Table S1). The mixed genomes having the highest ANI with most *Wolbachia* sequences belonging to supergroup A (Table 5). Combining the ANI result with the MLST ML tree (Fig. 1), we are confident that both *w*Jav1 and *w*Jav2 belong to supergroup A.

**Table 5:** The closest Average Nucleotide Identity (ANI) of *w*Jav1 and *w*Jav2 with fifteen *Wolbachia* strains

Assembly accession of reference sequence	Assembly name	ANI (%)	Host species	Strain	Supergroup
GCF_014129525.1	ASM1412952v1	99.0233	*Drosophila tropicalis*	*w*Tro	A
GCF_018690095.1	ASM1869009v1	98.977	*Drosophila simulans*	*w*Au	A
GCF_000742435.1	ASM74243v1	98.9697	*Drosophila recens*	*w*Rec	A
GCF_017896345.1	ASM1789634v1	98.9346	*Aedes aegypti*	GV_2018_2	A
GCF_017896245.1	ASM1789624v1	98.9343	*Aedes aegypti*	GV_2018_4	A
GCF_017896325.1	ASM1789632v1	98.9328	*Aedes aegypti*	GV_2018_7	A
GCF_016584425.1	ASM1658442v1	98.9322	*Drosophila melanogaster*	*w*Mel	A
GCF_017896365.1	ASM1789636v1	98.9262	*Aedes aegypti*	GV_2018_1	A
GCF_014129655.1	ASM1412965v1	98.9213	*Drosophila arawakana*	*w*Ara	A
GCF_017896285.1	ASM1789628v1	98.9204	*Aedes aegypti*	GV_2018_6	A
GCF_022343905.1	ASM2234390v1	98.8941	*Drosophila*	F7_A	
GCF_016584375.1	ASM1658437v1	98.8928	*Drosophila melanogaster*	*w*MelOctoless	A
GCF_021496215.1	ASM2149621v1	98.8845	*Aedes aegypti*	*w*Mel_wC45_F9	A
GCF_021496155.1	ASM2149615v1	98.8844	*Aedes aegypti*	*w*Mel_YK_2020	A
GCF_021496175.1	ASM2149617v1	98.8844	*Aedes aegypti*	*w*Mel_GV_2020	A

Note: Information on all *Wolbachia* genomes can be referred to Table S1

### Mobile genetic elements in *w* Jav1 *and w* Jav2

**Fig. 2:**
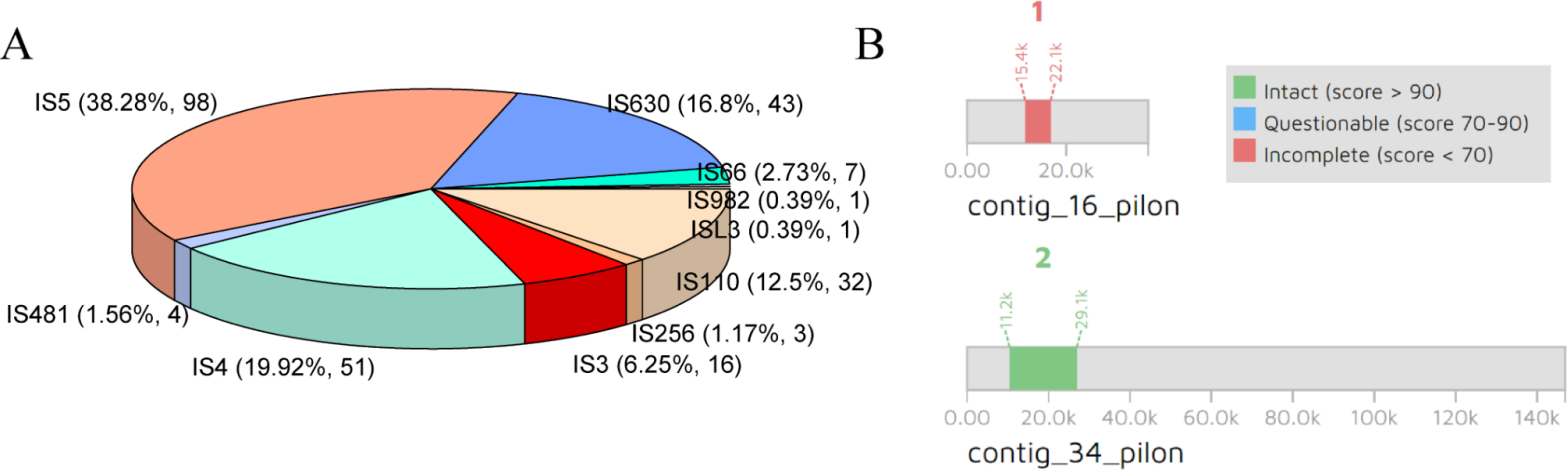
Summary of the mobile genetic elements situation in the mixed genomes of *w*Jav1 and *w*Jav2. **(A)** Proportion of ten main types of insertion sequence families. **(B)** Regions of two prophages referred to as WOjav1-2 in the genome (see Fig. 3 and Table S3 for details). The integrity of the prophage regions is marked by a different color

MGEs, mainly including prophage and insertion sequence (IS) in bacteria, are an important conduit for bacteria to acquire foreign genes and contribute to genome recombination. In the mixed genomes of *w*Jav1 and *w*Jav2, a total of two prophages (Fig. 2B) and 256 insertion sequences (Fig. 2A) were found, including 201 confirmed and 55 putative ISs.

**Fig. 3:**
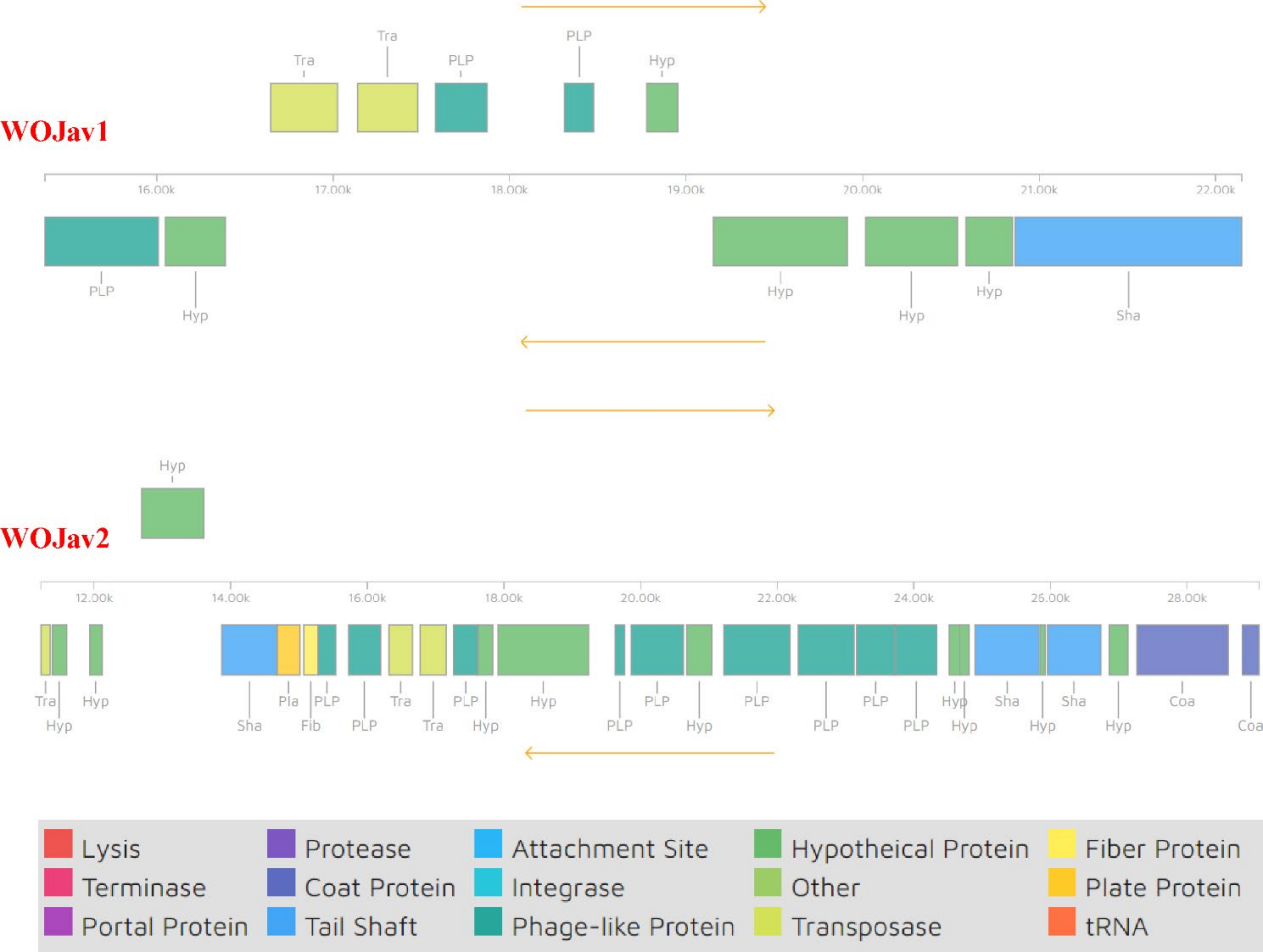
Prophage regions were detected in the mixed genomes of *w*Jav1 and *w*Jav2 using the PHASTER server. The number of genes discovered in each prophage region is shown in the lower panel with different colors. See Table S3 for details

Two prophages were as assigned the names WOjav1 and WOjav2, and most of the regions in them (Fig. 2B) are phage-like proteins, hypothetical proteins, and transposase proteins (Fig. 3; Table S3). WOjav1 is cryptic (structure incomplete) and only contains transposase proteins and minor tail proteins. However, WOjav2 is relatively intact, with complete phage replication and assembly related genes such as transposases, head morphogenesis proteins, baseplate assembly proteins with lysozyme, and tail proteins (Table S3).

The main types of ISs are similar to supergroup A *Wolbachia*, in that they are from the IS5, IS4, IS630, and IS110 families, which account for 38.28% (98), 19.92% (51), 16.8% (43), and 12.5% (32) of ISs, respectively. In the dominant IS family, there are two subgroups of IS5 named IS5_ssgr_IS903 (n = 43) and IS5_ssgr_IS1031 (n = 21) in 201 confirmed ISs. The IS families of the *Wolbachia* of *V. javana* from *F. hirta* are similar to the endosymbiont of the fig wasp *Ceratosolen solmsi* from *Ficus hispida* but numbers differ, 201 ISs for *V. javana* and 86 for *C. solmsi* [46].

### *Cif* genes in *w* Jav1 and *w* Jav2

**Fig. 4:**
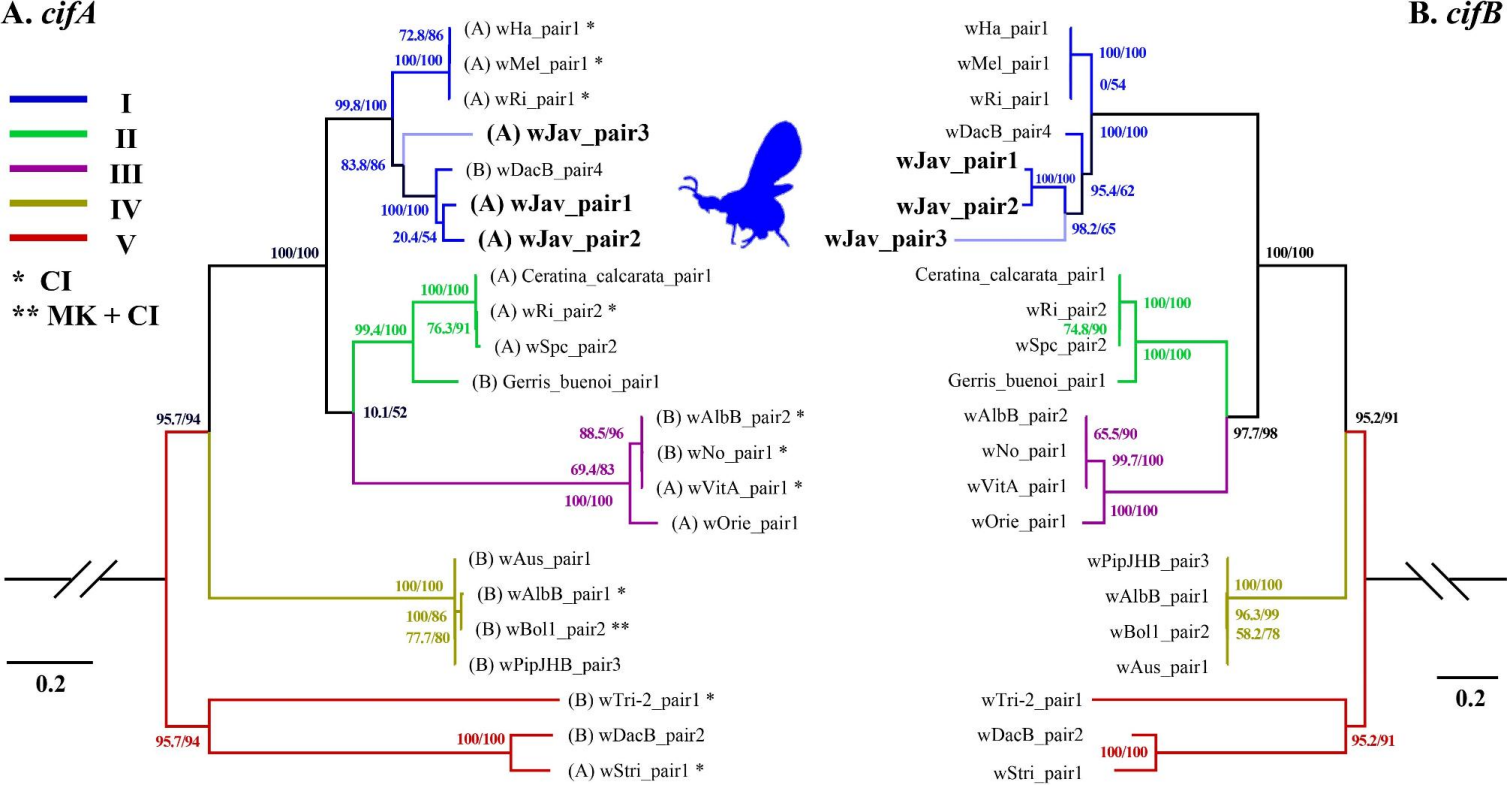
ML trees of **(A)** *cifA* and **(B)** *cifB* genes pairs in *w*Jav1-2 genomes and 19 *Wolbachia* strains. The SH-aLRT and UFBoot support values are given at the nodes. Supergroups of the *Wolbachia* strains are given in parentheses. * refers to CI (cytoplasmic incompatibility) host phenotype. ** refers to CI and MK (male killing). Corresponding colors that represent different *cif* types could be referred to Table S5. The corresponding strain names can be found in Table S6

In total, 4 proteins encoding genes were found to be aligned with two types (I and IV) of CifA, and 57 were aligned with three types (I, II, and V) of CifB in the *w*Jav1 and *w*Jav2 genomes (Table S4). The queried sequences fig|953.597.peg. 1691 (335 bp) and fig | 953.597.peg 1692 (1112 bp) may be the same *cifA* gene, but they are annotated as two genes because of multiple stop codons (Table S4). Therefore, the two genes were treated in tandem as one gene in the subsequent phylogenetic analysis. Fig | 953.597.peg 384 (1478 bp) and fig | 953.597.peg 1839 (1511 bp) are *cifA* genes (Table S4). The alignment length between fig|953.597.peg.995 and type V *cifB* is too short, and the alignment position is 3703aa − 3939aa (Table S4). Since this part is an ANK protein domain and is not a key conserved region of CifB, it was not analyzed further. Therefore, at least three different pairs of *cif* genes (*w*Jav_pair1-3 for short) were identified in the *w*Jav1 and *w*Jav2 genomes (Table S4). An ML tree involving *w*Jav_pair1-3 and other different types of *cif* genes (Table S5, Table S6 [20]) indicated that all *w*Jav_pair1-3 belong to type I, and *w*Jav_pair1-2 were particularly close and may be diverged from the same gene (Fig. 4).

### Genes related to biotin synthesis and metabolism in *w* Jav1 and *w* Jav2

**Fig. 5:**
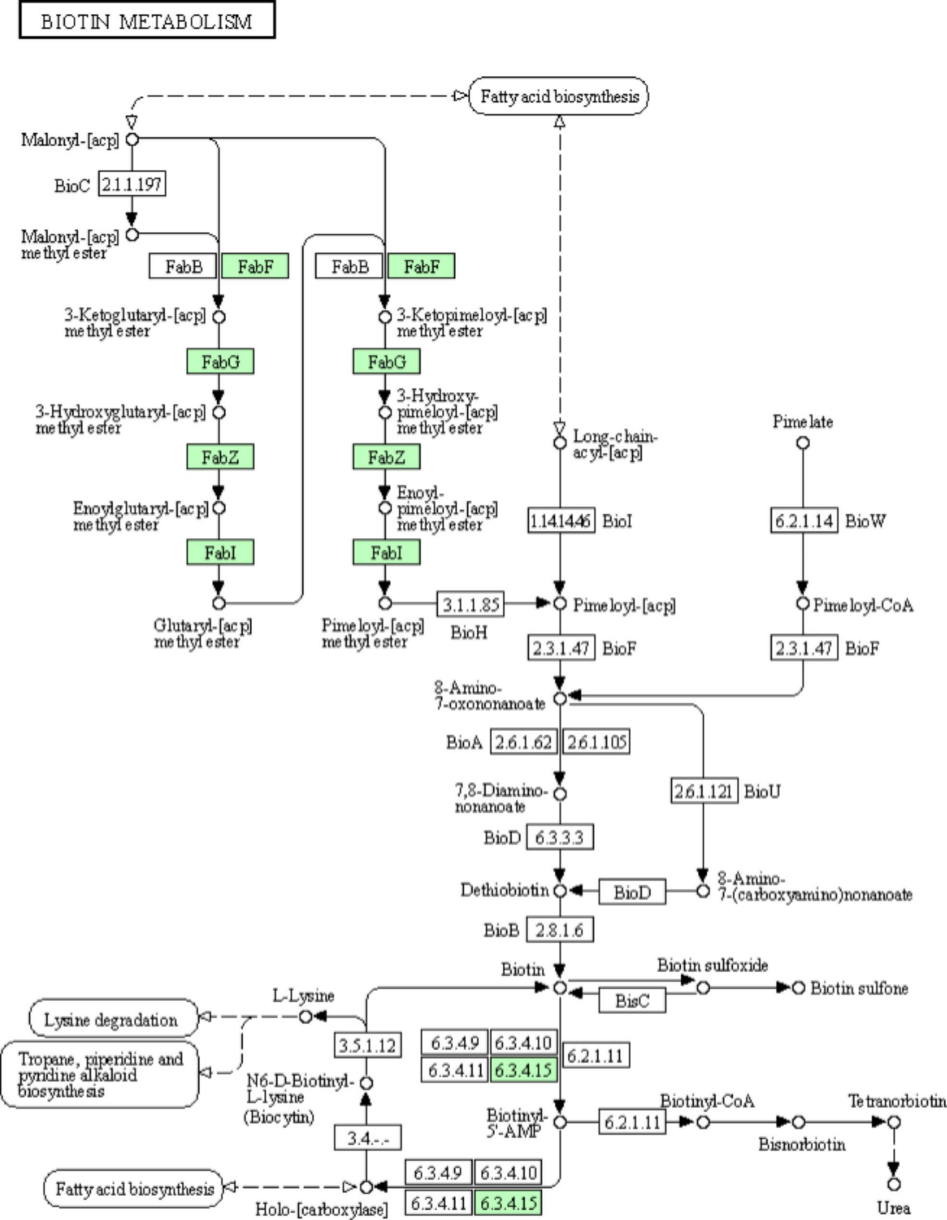
Schematic illustration of genes related to biotin synthesis and metabolism in *w*Jav1-2. The numbers in the box indicate the Enzyme Commission number. White and green boxes indicate the absence and presence of the corresponding enzyme, respectively, based on RAST annotations. See Table 6 for details

Based on the KEGG pathway enrichment analysis, the *w*Jav1 and *w*Jav2 mixed genomes do not contain any key genes (*BioF/A/D/B*) for biotin synthesis. However, the genes of glutaryl-[acp] methyl ester and pimeloyl-[acyl carrier protein] methyl ester, *fabF/G/Z/I*, which are the key precursors for the synthesis of biotin, and the genes *birA*, which metabolize biotinyl-5 ‘- AMP and holo-[carboxylase] downstream, are present (Fig. 5). The genome-wide functional annotation based on the RAST and eggnog web servers also indicated that the mixed genomes do not contain any key genes for the synthesis of biotin; however, the gene *bioY* for uptake of biotin from the host, and the gene *birA* that uses biotin as an essential precursor to synthesize their metabolites were present (Table 6).

**Table 6:** Genes related to biotin synthesis and metabolism were annotated in the *w*Jav genome

Gene ID	COG category	KEGG ko	PFAMs	Description	Preferred_name
Figure 953.597.peg.606	C	ko:K00627	2-oxoacid_dh, Biotin_lipoyl, E3_binding	The pyruvate dehydrogenase complex catalyzes the overall conversion of pyruvate to acetyl-CoA and CO(2)	*pdhC*
Figure 953.597.peg.285	C	ko:K00658	2-oxoacid_dh, Biotin_lipoyl, E3_binding	The 2-oxoglutarate dehydrogenase complex catalyzes the overall conversion of 2-oxoglutarate to succinyl-CoA and CO(2)	*sucB*
Figure 953.597.peg.373	M	ko:K02022,ko:K12542	Biotin_lipoyl_2, HlyD,HlyD_3	Secretion protein	*aprE*
Figure 953.597.peg.1008	M	ko:K02022,ko:K12542	Biotin_lipoyl_2, HlyD,HlyD_3	Secretion protein	*aprE*
Figure 953.597.peg.1587	H	ko:K03524	BPL_C, BPL_LplA_LipB, BPL_LplA_LipB_2	Biotin/lipoate A/B protein ligase family	*birA*
Figure 953.597.peg.1588	H	ko:K03524	BPL_C, BPL_LplA_LipB, BPL_LplA_LipB_2	Biotin/lipoate A/B protein ligase family	*birA*
Figure 953.597.peg.1204	I	ko:K01965	Biotin_carb_C, Biotin_carb_N, Biotin_lipoyl,CPSase_L_D2	Propionyl-CoA carboxylase alpha	*pccA*
Figure 953.597.peg.1314	C	ko:K00658	2-oxoacid_dh, Biotin_lipoyl, E3_binding	The 2-oxoglutarate dehydrogenase complex catalyzes the overall conversion of 2-oxoglutarate to succinyl-CoA and CO(2)	*sucB*
Figure 953.597.peg.2169	M	ko:K02022,ko:K12542	Biotin_lipoyl_2, HlyD,HlyD_3	Secretion protein	*aprE*
Figure 953.597.peg.2777	H	ko:K03524	BPL_C, BPL_LplA_LipB, BPL_LplA_LipB_2	Biotin/lipoate A/B protein ligase family	*birA*
Figure 953.597.peg.2495	H	ko:K03524	BPL_C,BPL_LplA_LipB,BPL_LplA_LipB_2	Biotin/lipoate A/B protein ligase family	*birA*
Figure 953.597.peg.2535	M	ko:K18990	Biotin_lipoyl_2, HlyD_D23	Belongs to the membrane fusion protein (MFP) (TC 8.A.1) family	
Figure 953.597.peg.637	S	ko:K03523	BioY	BioY family	*bioY2*

## Discussion

The high proportion of *Wolbachia* sequences in the PacBio data set for *V. javana* is indicative of a high *Wolbachia* titer. In contrast, *Wolbachia* can fail to proliferate in some wasp hosts. For example, as a maternal effect gene, *Wolbachia* identity suppressor (*Wds*) controls the titer of *Wolbachia* in *Nasonia* body and inhibits adverse effects from *Wolbachia* due to endosymbiont overproliferation [47]. Perhaps the high titer of *Wolbachia* in *V. javana* reflects a recent symbiotic relationship where control of the endosymbiont has not yet evolved, although there may also be a direct impact of diet composition of the pollinator that affects *Wolbachia* titers [48].

Two strains of *Wolbachia* from supergroup A appear to infect *V. javana* (Fig. 1, Table S1, Table S2). Other studies have shown that fig wasps could be infected by multiple strains of *Wolbachia*, and the strains of infected pollinators are mainly from supergroup A [9, 49]. Three possible scenarios may lead to the production of double infections: (i) *Wolbachia* are introduced into the same host multiple times, (ii) *Wolbachia* have diverged after invasion among lineages and are then transferred horizontally between lineages, and (iii) the common ancestor of *V. javana* was multiply infected, and this situation persisted during further divergence [50]. A double infection in a host may be maintained by synergism among different *Wolbachia* strains, but also through patterns of incompatibility produced through reproductive effects [51]. The presence of multiple strains can lead to a gene exchange between bacterial strains [52], and the strains can differ in density in a host [53].

The presence of multiple pairs of type I *cif* genes with three copies suggests that *w*Jav1 and *w*Jav2 are likely to induce strong CI. The MLST analysis indicates that related species of *V. javana* infected with *Wolbachia* are known to cause CI (Fig. 1). The closest endosymbiont in the *cif* gene ML tree was from *Dactylopius coccus* which has a type I *cif* (Fig. 3, Table S6) that leads to CI [54]. The *cif* genes belonging to type I generally cause and rescue CI (as shown in transgenic *Drosophila* and *Culex pipiens* [55] [56]). *Wolbachia* CI strength is related to *cifA*/*cifB* homolog similarity and copy number [18]. A strain with two or three copies such as *w*Ri and *w*Ha in *Drosophila simulans* induces strong CI [18]. Experiments are needed to test the prediction that strong CI is present in *V. javana*.

*Ficus hirta* is a dioecious plant, with far more female trees in the field than male trees [57]. The wingless male pollinator *V. javana* spend their whole life cycle in the figs of male trees (gall figs). The female wasps will fly out of gall figs after mating to spread *Wolbachia* and lay eggs in the gall flowers of other trees [58–61]. Female therefore spread *Wolbachia* through maternal transmission and the rate of spread will be enhanced by CI. When *Wolbachia*-uninfected females of *V. javana* lay eggs inside gall figs where *Wolbachia*-infected male pollinators are present, the offspring of the *Wolbachia*-uninfected females of *V. javana* will have a higher proportion of hatched males or eggs will fail to hatch. Studies have shown that both male and female pollinators of fig wasps can mate multiple times, although many females mate only once. In *Ceratoolen solmsi marchali*, females that multiply mate produce more female offspring, while the number of male offspring does not change significantly, resulting in a higher female sex ratio in offspring [62]. Combining this phenomenon with the reproductive regulation induced by *Wolbachia*, we speculate that after mating once, uninfected females will produce a male biased set of offspring (the oosperm dies, and the male figs wasps develops from the unfertilized egg). After multiple mating, the probability of females and male fig wasps uninfected by *Wolbachia* is high, resulting in an increase in the survival of females (oosperm). In addition, the high frequency of *Wolbachia* in populations may contribute to gender imbalance (female-biased sex ratios) of fig wasps [63]. However, experiments on infected and uninfected lines are required to test this further.

While *Wolbachia* from *V. javana* appears to have lost biotin-related genes, *Wolbachia* could still help the pollinators to absorb and metabolize biotin, which is synthesized by the host plant. As an essential vitamin for insects [64], biotin functions as a covalently-bound cofactor in various carboxylases, which have major roles in fatty acid biosynthesis, amino acid, and fatty acid catabolism, and also function in the citric acid cycle [65, 66].

To determine the distribution of biotin-related genes in the *Wolbachia*-pollinator-host plant system, we compared genome or transcriptome data from different fig tissues at the receptive phase (ostiolar bracts [67], female flowers [68], male flowers: unpublished), from the pollinator *V. javana* and from its endosymbiont *Wolbachia*. We found that biotin-synthesis genes in *F. hirta*, and the bract of the fig were more expressed (Table S7). The bracts, which are designed to attract insects, have additional biotin-related gene expression compared to female and male flowers of the male fig tree (Table S7). Female flowers, which are designed for pollination or oviposition, show the expression of *bioA*, *pdhC*, and *sucB* genes compared to male flowers (Table S7). Interestingly, *V. javana* has several biotin-related genes for absorbing and metabolizing biotin but far less than the endosymbiont (Table S7). Considering endosymbiont genes can be found in the host genome, a process of horizontal transfer from *Wolbachia* to the pollinator may have taken place [69]. Pollinators lay their eggs in the gall flowers, and their larvae feed on flowers with the biotin synthesis genes. Therefore, we speculate that pollinators acquire biotin from the gall flowers of *F. hirta*, and that *Wolbachia* helps the wasps to absorb and metabolize biotin.

Due to food with unbalanced nutrition and/or the absence of vitamin B, *Wolbachia* of *Bemisia tabaci* [70], planthoppers [71], and blood feeders maintain the function of synthesizing biotin. However, the *Wolbachia* of *V. javana* may have lost its biotin gene and *F. hirta* may provide a sufficiently balanced diet without the help of endosymbionts, or else *Wolbachia* may interact with different microorganisms (such as *Cardinium* [71]) which have a complete biotin synthesis pathway. Further studies including ecological observations, experimental studies, and molecular assessments are required to examine this further.

## Conclusions

We have assembled the mixed *Wolbachia* genomes *w*Jav1 and *w*Jav2 of *V. javana* constituting two novel *Wolbachia* strains belonging to supergroup A. The MLST analysis helped to understand the relatedness of these *Wolbachia* to others. The annotation and analysis of prophages and insertion sequences shows some features that are different from other fig wasp endosymbionts, and highlights potential effects on genome evolution and the gene content of intracellular symbiosis, and these elements could be reused as gene manipulation tools. The discovery of at least three pairs of *cifA*-*cifB* genes from prophages belonging to type I suggests CI in the pollinators of *F. hirta*, and provides a basis for further studies on reproductive effects. The *Wolbachia* genome may help to provide a further understanding of nutritional aspects in this system.

## Methods

### Genomes assembly of *Wolbachia*

We collected nearly 500–1,000 female adult samples of *V. javana* from several figs of *F. hirta* in a single tree on Baiyun Mountain in Guangdong Province, China. The *Wolbachia* genomes were assembled based on whole genome sequencing data (CNCB accession number of GWHBDGE00000000 for *V. javana*).

165 assembled versions of *Wolbachia* genomes from different host species downloaded by NCBI were used as the reference genome (Table S1). The PacBio raw data of the *V. javana* genome (coverage 40 ×; 11.9 GB) were aligned to the *Wolbachia* reference genome (Table S1) using BLASR v5.3.5 [72]. Illumina sequencing reads of *Wolbachia* were acquired by aligning the Illumina genome sequences of *V. javana* with the *Wolbachia* reference genomes using kneaddata v0.10.0 (https://huttenhower.sph.harvard.edu/kneaddata). Then *Wolbachia* PacBio sequencing reads in *V. javana* were assembled using Flye assembler v2.9-b1768 [73], and the resulting assembly polished in Pilon version 1.23 [74] with Illumina data.

**Fig. 6:**
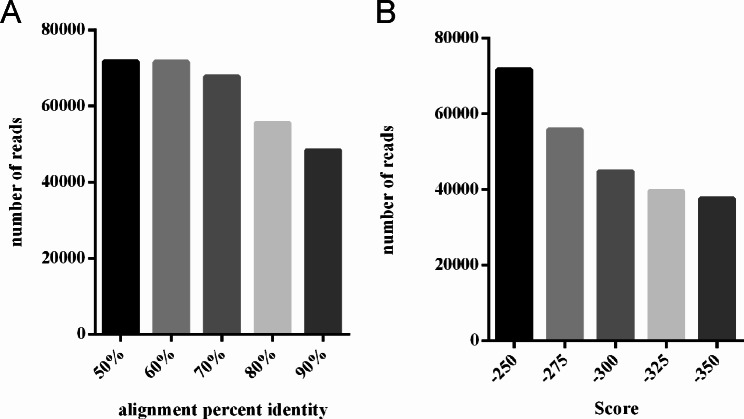
Number of *Wolbachia* reads aligned by BLASR in PacBio sequencing of the host *Valisia javana* genome. **(A)** Number of *Wolbachia* genome reads at different alignment percent identity. **(B)** Number of *Wolbachia* genome reads at different scores

The large number of *Wolbachia* genomic fragments in the sequence data of *V. javana* [43] was found. There were 861,951 reads, with a total length of 12,005.70 Mb and a coverage of about 40× to the *V. javana* genome (296.34 Mb). After using BLASR to set different identities and maxscore, different numbers of *Wolbachia* reads were obtained (Fig. 6A and B). We performed genome assembly using reads under different parameters, and the results showed that many sequences did not belong to *Wolbachia*, falling between the parameters maxscore − 250 and − 300 of the BLASR screen (Table 1). Using the BLASR parameters as Identity ≥ 60% and maxscore ≤ -250, the aligned reads were assembled, and the final genome size exceeded 50 Mb, which was inconsistent with the normal size of the *Wolbachia* genome (Table S1). Therefore, we finally used the BLASR parameters Identity ≥ 60% and maxscore ≤ -300 to assemble the PacBio sequencing data of about 610 Mb. According to the assembled *Wolbachia* longest genome of 2,352,936 bp, the coverage is more than 270×.

### Genome annotation

The genome size and GC content of the assembled *Wolbachia* were calculated with seqkit v0.9.2 [75]. Rapid genome annotation was conducted by RAST server (https://rast.nmpdr.org/rast.cgi). SEED Viewer v2.0 was used to view the KEGG pathway [76] through genomic results annotated by the RAST server. Insert sequences (ISs) were found in the IS database using ISsaga web server (http://issaga.biotoul.fr/issaga_index.php) [77]. The prophage regions in *Wolbachia* genome were annotated using Phaster web server (http://phaster.ca), and the visualization of the positions of the prophage regions was automatically generated by the Phaster web server. Functions and potentially involved pathways of the coding genes in *Wolbachia* genome were further annotated using eggnog Mapper web server (http://eggnog-mapper.embl.de) [78] and the KEGG automatic annotation server KAAS (https://www.genome.jp/tools/kaas/) [79].

### Identification and phylogenetic analysis of *Wolbachia* in *V. javana*

To identify the *Wolbachia* strains in *V. javana*, five multilocus sequence typing genes (MLST) including *gatB*, *fbpA*, *coxA*, *hcpA*, and *ftsZ* in genomic data were blasted by BLASTN v2.9.0 according to the reference *w*Mel strain of *Drosophila melanogaster* whose sequence type is typical ST1 (Table S2). The sequence type of the *Wolbachia* in *V. javana* was identified by aligning with PubMLST *Wolbachia* MLST database (http://www.pubmlst.org/Wolbachia/). In addition, ST sequences (sequences synthesized by housekeeping genes) of MLST in other *Wolbachia* genomes of A, B, D, F, and H supergroups were downloaded from PubMLST *Wolbachia* MLST database (Table S2).

Multiple sequence alignments for these MLST genes were aligned by MAFFT v7.310 [80]. The most suitable substitution model was calculated by ModelFinder [81], and the maximum-likelihood tree was constructed by IQ-tree v1.6.10 [82] with GTR + F + I + G4 model chosen according to BIC, and SH-aLRT test and ultrafast bootstrap with 1000 replicates. MLST genes of *Wolbachia* strain Zang_H of dampwood termites *Zootermopsis angusticollis* is selected as the outgroup (Table S2). In addition, the average nucleotide similarity (ANI) between *Wolbachia* genomes was calculated using fastANI v1.33 to further quantify the relationship between this genome and other *Wolbachia* genomes (Table S1) [83].

### Identification of *cif* genes

Based on the typical CifA and CifB protein sequences of type I-V (Table S5), the *cif* genes and their homologous in *Wolbachia* of *V. javana* were blasted by blastp v2.9.0. The selection of homologues was based on Lindsey’s method [19] and slightly improved by the following criteria: (1) e ≤ 10^− 30^; (2) a percentage of identity matches ≥ 50%. The obtained *cif* genes were used to construct the ML tree with the *cif* genes from other *Wolbachia* strains [20]. Model (TPM3 + F + G4) and Bootstraps (SH-aLRT test and ultrafast bootstrap with 1000 replicates) settings are the same for *cifA* and *cifB*. *Cif* genes of *wTri*-2_pair1, *wDacB*_pair2, and *wStri*_pair1 were selected as outgroups (Table S6).

### Electronic supplementary material

Below is the link to the electronic supplementary material.

Supplementary Material 1

## Data Availability

Data for this study will be completed after the manuscript is accepted for publication. All genome sequence data of *Wolbachia* are available in GWH database of the National Genomics Data Center (NGDC) with accession no. GWHCBHN00000000. The raw genome sequencing data of Illumina and PacBio have been deposited in the GSA database of the National Genomics Data Center (NGDC) with accession number of CRA010785. All result data were deposited in Figshare (doi: 10.6084/m9.figshare.23553021).
